# Factors Associated With Protection From SARS-CoV-2 Omicron Variant Infection and Disease Among Vaccinated Health Care Workers in Israel

**DOI:** 10.1001/jamanetworkopen.2023.14757

**Published:** 2023-05-23

**Authors:** Mayan Gilboa, Tal Gonen, Noam Barda, Shelly Cohn, Victoria Indenbaum, Yael Weiss-Ottolenghi, Sharon Amit, Keren Asraf, Gili Joseph, Tal Levin, Yara Kanaaneh, Alex Aydenzon, Michal Canetti, Laurence Freedman, Neta Zuckerman, Ella Mendelson, Ram Doolman, Yitshak Kreiss, Gili Regev-Yochay, Yaniv Lustig

**Affiliations:** 1 The Sheba Pandemic Preparedness Research Institute, Sheba Medical Center, Ramat Gan, Israel; 2 The Infection Prevention & Control Unit, Sheba Medical Center, Ramat Gan, Israel; 3 Sackler School of Medicine, Tel-Aviv University, Tel Aviv, Israel; 4 ARC Innovation Center, Sheba Medical Center, Ramat Gan, Israel; 5 Software and Information Systems Engineering, Ben-Gurion University of the Negev, Be’er Sheva, Israel; 6 Department of Epidemiology, Biostatistics and Community Health Sciences, Ben-Gurion University of the Negev, Be’er Sheva, Israel; 7 Central Virology Laboratory, Public Health Directorate, Ministry of Health, Sheba Medical Center, Ramat Gan, Israel; 8 Clinical Microbiology, Sheba Medical Center, Ramat Gan, Israel.; 9 The Dworman Automated-Mega Laboratory, Sheba Medical Center, Ramat-Gan, Israel; 10 Biostatistics and Biomathematics Unit, Gertner Institute of Epidemiology and Health Policy Research, Sheba Medical Center, Ramat-Gan, Israel; 11 General Management, Sheba Medical Center, Ramat Gan, Israel

## Abstract

**Importance:**

A correlation between antibody levels and risk of infection has been demonstrated for the wild-type, Alpha, and Delta SARS-COV-2 variants. High rates of breakthrough infections by the Omicron variant emphasized the need to investigate whether the humoral response elicited by mRNA vaccines is also associated with reduced risk of Omicron infection and disease.

**Objective:**

To investigate whether the high antibody levels in individuals who have received at least 3 doses of an mRNA vaccine are associated with reduced risk of Omicron infection and disease.

**Design, Setting, and Participants:**

This prospective cohort study used serial real time–polymerase chain reaction (RT-PCR) and serological test data from January and May 2022 to assess the association of preinfection immunoglobin G (IgG) and neutralizing antibody titers with incidence of Omicron variant infection, incidence of symptomatic disease, and infectivity. Participants included health care workers who had received 3 or 4 doses of an mRNA COVID-19 vaccine. Data were analyzed from May to August 2022.

**Exposures:**

Levels of SARS-CoV-2 anti–receptor binding domain IgG and neutralizing antibodies.

**Main Outcomes and Measures:**

The main outcomes were incidence of Omicron infection, incidence of symptomatic disease, and infectivity. Outcomes were measured using SARS-COV-2 PCR and antigen testing and daily online surveys regarding symptomatic disease.

**Results:**

This study included 3 cohorts for 3 different analyses: 2310 participants were included in the protection from infection analysis (4689 exposure events; median [IQR] age, 50 [40-60] years; 3590 [76.6%] among female health care workers), 667 participants (median [IQR] age, 46.28 (37.44,54.8); 516 [77.4%] female) in the symptomatic disease analysis, and 532 participants (median [IQR] age, 48 [39-56] years; 403 [75.8%] female) in the infectivity analysis. Lower odds of infection were observed for each 10-fold increase in preinfection IgG (odds ratio [OR], 0.71; 95% CI, 0.56-0.90) and for each 2-fold increase in neutralizing antibody titers (OR, 0.89; 95% CI, 0.83-0.95). The odds of substantial symptomatic disease were reduced for each 10-fold increase in IgG levels (OR, 0.48; 95% CI, 0.29-0.78) and for each 2-fold increase in neutralizing antibodies levels (OR, 0.86; 95% CI, 0.76-0.96). Infectivity, assessed by mean cycle threshold value, was not significantly decreased with increasing IgG or neutralizing antibodies titers.

**Conclusions and Relevance:**

In this cohort study of vaccinated health care workers, IgG and neutralizing antibody titer levels were associated with protection against infection with the Omicron variant and against symptomatic disease.

## Key Points

**Question:**

Are antibody levels associated with protection against infection with the SARS-COV-2 Omicron variant?

**Findings:**

This cohort study that included 2310 adults found that the odds of infection and of substantial symptomatic disease significantly decreased for each 10-fold increase in preinfection immunoglobin G titers and for each 2-fold increase in neutralizing antibody titers.

**Meaning:**

These findings suggest that antibody titers were associated with protection against infection with the Omicron variant of SARS-CoV-2 and against symptomatic disease once infected.

## Teaser

This cohort study assesses associations of levels of humoral response with protection from SARS-CoV-2 Omicron infection, symptomatic disease, and infectivity among vaccinated health care workers in Israel.

## Introduction

Previous studies have reported an association between antibody titers and protection from infection with SARS-CoV-2 at both the individual and population level.^1,2,3^ However, those studies were conducted before the emergence of the highly infectious Omicron variant of concern (VOC), and enrolled individuals who were vaccinated with 2 vaccine doses, before a third vaccine dose was widely recommended.

The Omicron VOC emerged in Israel in late 2021, along with the waning of the humoral response that followed the third vaccine dose.^4^ The dramatic increase in breakthrough infections among vaccinated individuals drove decision-makers in Israel to recommend the administration of a fourth BNT162b2 (Pfizer/BioNTech) vaccine dose to health care workers (HCWs) and other populations considered at high risk, such as older adults and people with immune disorders. This vaccination campaign was associated with protection against mortality and hospitalizations among those vaccinated.^5^ However, the efficacy against infection was not as marked as it was found to be for previous doses,^6^ and the appropriate timing of the fourth dose in the general population remained unknown.

In this study, we investigated whether humoral immunity can serve as a biomarker associated with protection against infection and symptomatic disease from SARS-CoV-2 Omicron VOC, at a time when the BA.1 and BA.2 lineages were the most predominate infections in Israel. Specifically, we explore the association of an individual’s preinfection titers of antispike immunoglobin G (IgG) and neutralizing antibodies with 3 different outcomes: SARS-CoV-2 infection (as confirmed by polymerase chain reaction [PCR] testing), symptomatic disease, and infectivity among those infected.

## Methods

### Study Setting and Design

Sheba Medical Center (SMC) is Israel’s largest tertiary Medical Center, with more 15 000 HCWs. All HCW who received vaccination for SARS-CoV-2 in the medical center were invited to participate in this study, and all HCWs who agreed to participate signed a written informed consent form. This cohort study was approved by the institutional review board at SMC. This study is based on the SMC HCW COVID cohort, an ongoing prospective cohort study following SMC personnel via virologic and serological testing that began in December 2020, with the initiation of the BNT162b2 vaccination campaign in Israel, as has been previously described in detail.^1,4,6,7,8,9,10,11^ This study is reported following the Strengthening the Reporting of Observational Studies in Epidemiology (STROBE) reporting guideline.

All participants received at least 3 doses of the BNT162b2 COVID-19 vaccine. Study participants were tested monthly for serologic markers and were reminded to do so via email, text messages, and telephone reminders, sent periodically to increase adherence. At recruitment, all participants were sent a questionnaire regarding their medical history (eTable 1 in Supplement 1). This study began in January 2022, when the Omicron VOC became dominant in Israel, and ended in May 2022, as infection rates with SARS-CoV-2 waned. During those months, a significant surge of BA.1, followed by BA.2 lineages, took place in Israel. All SMC HCWs who had test results positive for SARS-CoV-2 between January 1, 2022, and May 3, 2022, were sent daily online surveys regarding the symptoms they experienced during their mandatory 5-day isolation period as well as a medical history questionnaire to account for changes from the baseline questionnaire (eTable 2 in Supplement 1). HCWs were able to undergo PCR tests for SARS-CoV-2 in a designated clinic at SMC. Nasopharyngeal swabs were collected by trained personnel and analyzed by quantitative real-time PCR via the Allplex 2019-nCoV platform (Seegene). As part of a surveillance program of the Israeli Ministry of Health, SARS-CoV-2–positive samples from the community as well as from SMC were randomly selected for sequencing, which was performed using the COVIDSeq kit for library preparation and Illumina platforms (Illumina).

### PCR-Confirmed SARS-CoV-2 Infection

The confirmed SARS-CoV-2 infection analysis compared antibody levels between individuals whose PCR test results were positive for SARS-CoV-2 vs those whose PCR test results were negative. The study population included all HCWs who performed a PCR test for SARS-CoV-2 at SMC during the study period. We clustered tests into exposure events, each defined as a cluster of PCR tests lasting no more than 5 days between the first (the start of the event) and the last test performed. Testing was primarily driven by exposure in the community or at work, but also by occurrence of symptoms. An event was defined as positive (a case) or negative (a control) by the last test in the cluster. Inclusion criteria for this analysis included having received at least 3 vaccine doses, performance of a serology test 3 to 30 days before the exposure event, and having no known history of SARS-CoV-2 infection (eMethods 1 in Supplement 1).

### Symptomatic Disease

The symptomatic disease analysis compared the incidence of symptomatic disease at varying severity among individuals with varying preinfection antibody titers. The unit of observation was a single individual. Eligibility criteria for this analysis included a positive SARS-CoV-2 PCR or antigen-detecting rapid diagnostic test (Ag-RDT) result, no known history of SARS-CoV-2 infection, having answered at least 1 symptom survey, and performance of a serology test 3 to 30 days before the positive PCR test result (eMethods 2 in Supplement 1).

Outcomes of interest included substantial symptomatic disease (binary, defined as ≥1 day of fever >38 °C or ≥1 day of systemic symptoms necessitating complete bed rest), the duration of substantial symptomatic disease (ordinal), fever greater than 38 °C (binary), fever duration (ordinal), and any symptomatic disease (binary).

### Infectivity

The infectivity analysis examined the association between preinfection antibody titers and the cycle threshold (Ct) values for the gene encoding the nucleocapsid protein (N gene), measured by PCR testing. The unit of observation was an individual’s first positive PCR test result. Eligibility criteria for this analysis included a positive PCR test result, no known history of SARS-CoV-2 infection, having received at least 3 doses of an mRNA COVID-19 vaccine, and the performance of a serology test 3 to 30 days prior to the positive PCR test result. All study variables, including their use and definitions, are detailed in eTable 1 in Supplement 1.

### Exposures

In each analysis, the exposures of interest were levels of SARS-CoV-2 anti–receptor binding domain (RBD)IgG and neutralizing antibodies. Testing for IgG titers was conducted using the SARS-CoV-2 IgG II Quant (6S60; Abbott) kit according to the manufacturer’s instructions, and testing for neutralizing antibody titers by a SARS-CoV-2 pseudovirus (psSARS-2) neutralization assay, using a vesicular stomatitis virus–based wild-type (WT) SARS-CoV-2 spike.^7^ Further details on kits used and methods are provided in eMethods 3 in Supplement 1.

### Statistical Analysis

In each model, both exposures (IgG and neutralizing antibody levels) were log-transformed, using base-10 for IgG and base-2 for neutralizing antibodies. For the infection analysis, we first compared the estimated geometric means of antibody levels (with their 95% CIs) between infection and noninfection events. We then discretized IgG (0-500, 501-1000, 1001-2000, and ≥2001) and neutralizing (0-512, 1024, 2048-4096, and 8192) antibody levels and estimated the crude odds ratios (ORs) of infection at each level compared with the lowest one. Finally, we used a logistic regression model to estimate the adjusted ORs (aORs) of the associations between antibody levels and infection. The analysis adjusted for age, sex, number of COVID-19 vaccines received, time since last vaccination, and calendar time (eTable 1 in Supplement 1), with the logarithm of the antibody levels entered as a continuous variable, calendar time modeled using a restricted cubic spline, and a random effect used to account for repeated measurements at the level of the individual.

Symptomatic disease was studied similarly. We first estimated the geometric mean titers (GMTs) of antibody levels (with their 95% CIs) among individuals who did and did not experience the different outcomes. We then discretized the antibody levels (IgG: 1-500, 501-900, 901-1600, and ≥1600; neutralizing antibodies: 1-256, 512-1024, 2048, and 4096) and estimated the crude OR of each outcome at each level compared with the lowest antibody level. Finally, adjusted associations were estimated using logistic regression (substantial symptomatic disease, fever >38 °C, and symptomatic disease), negative binomial regression (number of days of substantial disease and number of days of fever), and ordinal logistic regression (maximum temperature measured), adjusting for age, sex, number of COVID-19 vaccines received, time since last vaccination, and the number of comorbidities. In each case, we report the exponentiated coefficient and its 95% CI, with the interpretation per the model used.

Infectivity was studied by plotting Ct values as a function of the antibody levels and overlaying a crude linear regression line with its 95% CI. Adjusted estimates were derived using multivariable linear regression, adjusted for age, sex, number of COVID-19 vaccines received, and the time since the last vaccine was received.

Missing data only existed in the analysis for symptomatic disease, where they were handled by imputing the missing data 5 times, fitting the model on each imputed data set, and pooling the estimates using Rubin rules. Continuous variables were imputed by predictive mean–matching and binary variables by logistic regression.

Analyses were conducted using R version 4.1.2 (R Project for Statistical Computing). *P* values were 2-tailed, and statistical significance was set at 5%. Data were analyzed from June 2022 to April 2023.

## Results

**Figure 1.**
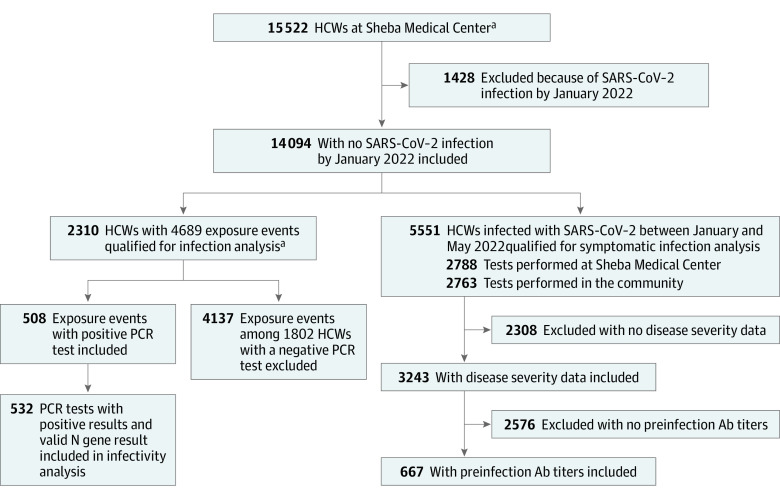
Study Design and Population Study design of the Sheba Medical Center health care workers (HCW) cohort following vaccination and infection with the Omicron variant. Ab indicates antibody. ^a^Previously uninfected HCW who received at least 3 doses of the BNT162b2 vaccine, performed at least 1 SARS-CoV-2 polymerase chain reaction (PCR) test at Sheba Medical Center and had undergone at least 1 serology test 3 to 30 days prior to PCR testing.

Between January 2022 and May 2022, 5551 of 14 094 previously uninfected HCWs were infected with SARS-CoV-2. Of them, 2788 were diagnosed in SMC (by PCR) and 2763 were diagnosed in the community, by either PCR or Ag-RDT. This study included 3 cohorts for 3 different analyses: 2310 participants were included in the protection from infection analysis (4689 exposure events; median [IQR] age, 50 [40-60] years; 3590 [76.6%] among female HCWs), 667 participants (median [IQR] age, 46.28 (37.44,54.8); 516 [77.4%] female) in the symptomatic disease analysis, and 532 participants (median [IQR] age, 48 [39-56] years; 403 [75.8%] female) in the infectivity analysis (Figure 1). Baseline characteristics of these populations are presented in Table 1 and eTable 3 and eTable 4 in Supplement 1.

**Table 1.** Characteristics of the Protection From Infection Study Population at Their Exposure Event

Factor	Events, No. (%)		
	All included in the analysis (n = 4689)^a^	That did not result in an infection (n = 4137)	That resulted in an infection (n = 552)
Age, median (IQR), y	50 (40-60)	50.8 (41.3-60.7)	47.7 (39.0-56.5)
Sex			
Male	1099 (23.4)	964 (23.3)	135 (24.5)
Female	3590 (76.6)	3173 (76.7)	417 (75.5)
Received 4 vaccine doses during the study period	1453 (31.0)	1330 (32)	123 (22.3)
Time from last vaccine dose, median (IQR), d	123.00 (44.00-152.00)	116 (42-150)	147 (106.5-162)
Time from serology test to exposure event, median (IQR), d	14 (8-22)	14 (8-22)	14 (8-21)
IgG levels, BAU			
0-500	1031 (22.0)	886 (21.4)	145 (26.3)
501-1000	1158 (24.7)	998 (24.1)	160 (29)
1001-2000	1161 (24.8)	1018 (24.6)	143 (25.9)
>2000	1339 (28.6)	1235 (29.9)	104 (18.8)
Neutralizing antibody titer			
0-512	924 (35.2)	753 (33.3)	171 (47.2)
1024	530 (20.2)	449 (19.9)	81 (22.4)
2048-4096	847 (32.3)	760 (33.6)	87 (24)
>4096	321 (12.2)	298 (13.2)	23 (6.4)

^a^ A health care worker could contribute more than 1 event that did not result in an infection but was censored following their first infection.

During the study period, the dominant VOCs in Israel were Omicron BA.1 and BA.2. Although we did not establish a lineage for all samples from HCW with SARS-CoV-2 infection, sequencing was performed on 74 819 samples that were obtained during the study period from all over Israel and showed that 99.7% of the isolated strains were of the Omicron VOCs; among them, 58.9% were BA.1, 38.7% were BA.2, and 1.7% were BA4/5. The Delta VOC comprised the remaining 0.3% of samples (eFigure 1 in Supplement 1).

### PCR-Confirmed SARS-CoV-2 Infection Analysis

During the study period, 2310 HCWs contributed 4689 exposure events to the analysis, and 552 events resulted in SARS-COV-2 infections (Figure 1A). Baseline and demographic characteristics of participants contributing to these events are shown in Table 1.

**Figure 2.**
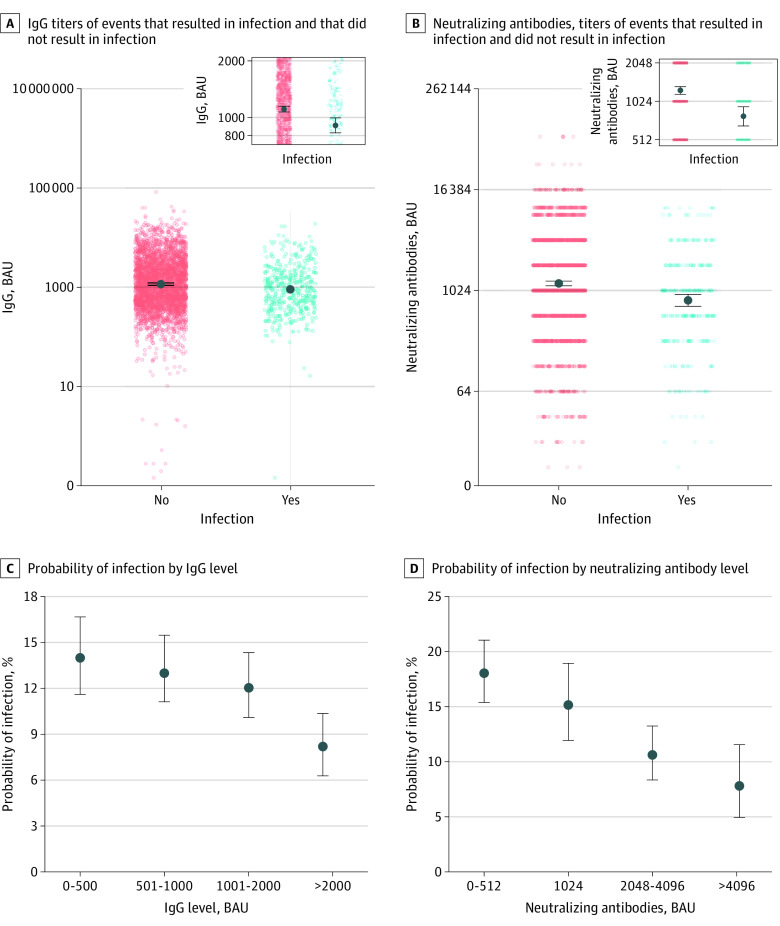
Associations of Antibody Titers and Confirmed SARS-CoV-2 Infection All infections were confirmed with polymerase chain reaction testing. Immunoglobin G (IgG) are presented as log-10; neutralizing antibodies, log-2.

Crude antibody titers at baseline were higher in HCWs who contracted SARS-CoV-2 infection than in those who did not. The GMT of IgG was 1118 (95% CI, 1083-1155) BAU in individuals with SARS-CoV-2 infection and 895 (95% CI, 825-969) BAU individuals who were not infected. GMTs of neutralizing antibody titers were 1213 (95% CI, 1142-1288) in individuals with SARS-CoV-2 infection, compared with 751 (95% CI, 649-899) in those who were not infected (Figure 2A and B). HCWs with preinfection IgG levels greater than 2000 BAU were less likely to become infected than those with IgG levels equal to or less than 500 BAU (OR, 0.52; 95% CI, 0.39-0.67). We observed lower odds of infection for participants with baseline neutralizing antibody titers between 2048 and 4096 (OR, 0.50; 95% CI, 0.38-0.66) and those with antibody titers greater than 4096 (OR, 0.34; 95% CI, 0.21-0.52), compared with participants with neutralizing antibody titers less than 1024 (Figure 2C and D; eTable 5 in Supplement 1).

A multivariable analysis adjusting for age, sex, receipt of a fourth vaccine dose, time from last vaccination, and calendar time estimated that higher IgG and neutralizing antibody titers were associated with a reduced probability of infection. For IgG titers, each 10-fold increase in antibody titers was associated with nearly 30% lower odds of infection (aOR, 0.71; 95% CI, 0.56-0.90). For neutralizing antibody titers, each 2-fold increase in antibody titers was associated with 11% reduced odds of infection (aOR, 0.89; 95% CI, 0.83-0.95) (Table 2; eTable 6 in Supplement 1).

**Table 2.** Summary of Regression Models for Association of Antispike IgG and Neutralizing Antibody Titers With Infection, Infectivity, and Symptomatic Disease

Outcome	aOR (95% CI)	
	Log10 IgG	Log2 Neutralizing antibodies
PCR-confirmed SARS-CoV-2 infection^a^	0.71 (0.56 to 0.90)	0.89 (0.83 to 0.95)
Infectivity^b^	1.25 (−0.11 to 2.62)	0.20 (−0.14 to 0.55)
Symptomatic disease^c^		
Substantial symptomatic disease	0.48 (0.29 to 0.78)	0.86 (0.76 to 0.96)
Duration of substantial symptoms, per d	0.82 (0.6 to 1.12)	0.94 (0.87 to 1.02)
Any symptomatic disease	0.72 (0.29 to 1.80)	0.81 (0.64 to 1.03)

Abbreviations: aOR, adjusted odds ratio; IgG, immunoglobin G.

^a^ Covariates for adjustment included age, sex, number of COVID-19 vaccines received, time since last vaccination, and calendar time.

^b^ Covariates for adjustment included age, sex, number of COVID-19 vaccines received, and time since last vaccination. Data presented as change in expected mean cycle threshold (95% CI) per 10-fold IgG or 2-fold neutralizing antibody change.

^c^ Covariates for adjustment included age, sex, number of COVID-19 vaccines received, time since last vaccination, and the number of comorbidities.

### Symptomatic Disease Analysis

Data on self-reported disease severity were available for 3243 of 5551 HCWs with Omicron infections (58%) (Figure 1A). eTable 7 in Supplement 1 presents the baseline characteristics of survey responders compared with nonresponders. The 2 populations were similar in age, sex, and vaccination history. eTable 8 in Supplement 1 summarizes the symptoms that HCWs with Omicron infections reported experiencing. Of those who responded to the symptom survey, preinfection IgG titers were available for 667 HCWs, and preinfection neutralizing antibody titers were available for 458 HCWs. Characteristics of HCWs included in this analysis are shown in eTable 3 in Supplement 1.

**Figure 3.**
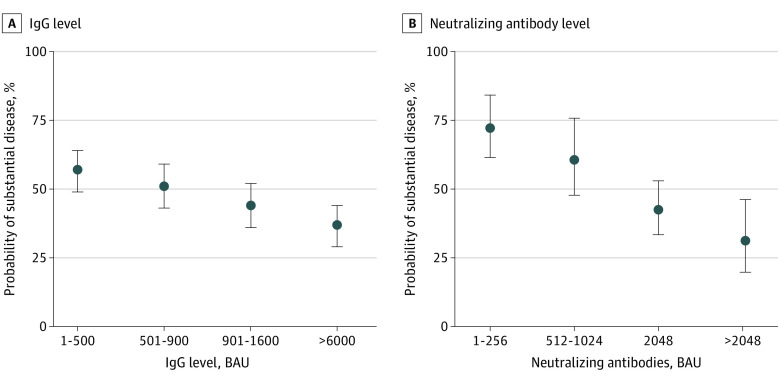
Associations of Antibody Titers and Substantial Symptomatic Disease Substantial symptomatic disease was defined as at least 1 day of fever greater than 38 °C or at least 1 day of systemic symptoms necessitating complete bed rest. IgG indicates immunoglobin G.

Odds of substantial symptomatic disease were lower among HCWs with preinfection IgG levels between 901 and 1600 BAU (OR, 0.59; 95% CI, 0.38-0.90) or greater than 1600 BAU (OR, 0.43; 95% CI, 0.28-0.66), compared with HCWs with preinfection IgG levels equal to or less than 500 BAU. Odds of substantial symptomatic disease were lower among participants with neutralizing antibody titers of 2048 (OR, 0.48; 95% CI, 0.26-0.89) or greater than 2048 (OR, 0.38; 95% CI, 0.21-0.67), compared with HCWs with titers less than 512 (Figures 3A and B; eTable 9 in Supplement 1). eFigure 2 in Supplement 1 presents the associations of crude antibody titers (IgG and neutralizing antibodies) and 2 outcomes: duration of substantial symptoms and fever (>38 °C) reported in symptom surveys.

In a negative binomial regression model that adjusted for age, sex, the number of comorbidities, receipt of a fourth vaccine dose, and time since last vaccination, the odds of substantial symptomatic disease were more than 50% lower for each 10-fold increase in IgG levels (aOR, 0.48; 95% CI, 0.29-0.77), and nearly 15% lower for each 2-fold increase in neutralizing antibody levels (aOR, 0.86; 95% CI, 0.76-0.96). Further results are presented in eTable 10 in Supplement 1.

### Infectivity Analysis

Overall, 532 individuals with SARS-CoV-2 infection were eligible for inclusion infectivity analysis. Baseline characteristics of participants in the infectivity analysis are presented in eTable 4 in Supplement 1. eFigure 3 in Supplement 1 presents the crude values of the N gene Ct results.

In a multivariable analysis adjusted for age, sex, receipt of the fourth vaccine dose, and time since last vaccination, there was no significant association between infectivity and each 10-fold increase in IgG (mean Ct, 1.25; 95% CI, −0.11 to 2.62) or each 2-fold increase in neutralizing antibody titers (mean Ct, 0.20; 95% CI, −0.14, 0.55) (Table 2; eTable 11 in Supplement 1).

## Discussion

In this large prospective cohort study, we found in adjusted analyses that higher titers of antispike IgG and neutralizing antibodies were associated with decreased risk for SARS-CoV-2 Omicron infection and milder symptoms among those who were infected. Importantly, we were able to determine infection and symptomatic disease ratios at varying antibody titers and the linear association of higher IgG and neutralizing antibody titers with a reduced probability of infection and symptomatic disease caused by an infection with the Omicron VOC. Immunological studies serve an important role in decision-making for regulatory agencies’ guidance and approval of new variant-specific vaccines and boosters, extending approval of vaccines to younger age groups, and decisions regarding coadministrations with other vaccines.^12^ A critical priority is to define factors associated with protection for new variants to facilitate identification of individuals who are at the most risk and who would benefit most from additional or booster doses. Several studies have found that peri-infection antibody titers were associated with protection from infection with previous variants (ie, wild-type, Alpha, and Delta)^1,7,13,14,15,16,17^; however, whether antibody titers are also associated with protection against infection with the Omicron VOC and, even more importantly, against substantial symptomatic disease caused by Omicron, is not fully understood.

Our results suggest that both IgG and neutralizing antibodies were associated with protection from infection and self-reported disease severity, independently from the receipt of a fourth vaccine dose and time since the last vaccination. Based on crude antibody levels, a multivariable analysis, and Poisson regression model, we found a dose-dependent association of antibody levels with protection from infection and substantial symptomatic disease. Reduced odds of PCR-confirmed infection (symptomatic or asymptomatic) were observed, albeit only at high IgG (>2000 BAU) and neutralizing antibody (>1024 BAU) titers, which is approximately 4 times higher than those needed to protect against the Delta variant.^17^ These results may explain the increase in vaccine effectiveness (VE) seen after a fourth vaccine dose^6,18^ and the decrease in VE as time passes from this dose.^5^ Based on our results and previous literature,^5,6,17,18^ we hypothesize that the fourth vaccine dose generates a rapid increase in antibody titers that may provide some initial protection, but protection gradually wanes as antibody levels decline. However, the introduction of bivalent vaccines targeted against both ancestral and Omicron SARS-CoV-2 could alter this decay in VE via an enhanced humoral response and increased protection against novel Omicron-related lineages, as was shown for both BA.1 and BA.5 bivalent mRNA vaccines.^19,20,21^ Hybrid immunity may also provide more durable protection against future Omicron infections due to induction of a greater humoral response that follows prior infection and vaccination, compared with vaccination alone.^22,23^

We did not find a significant association between preinfection antibody titers and infectivity, in contrast to our findings for the SARS-CoV-2 Alpha VOC.^1^ This could be explained in part due to the higher infectivity rate of the Omicron VOC, which is less affected by an individual’s serological status, or due to the dynamic nature of viral loads during the course of infection, which precludes direct assessment.

Our cohort of HCWs who received 3 or 4 vaccine doses allowed us to assess the possible associations among number of vaccine doses received, time since last vaccination, and IgG and neutralizing antibody levels. Our multivariable model showed that serological markers were associated with protection from infection independently from booster status and time since the last vaccination, suggesting that they are an essential component in protection from infection and not merely markers in this case.

### Limitations

This study has several limitations; first, we only tested for antispike IgG and neutralizing antibodies against the WT strain and not for specific anti-Omicron BA.1 or BA.2 antibodies. Our finding that infection and severity of disease with the Omicron VOC were associated with titers of antibodies directed against the WT strain suggests that titers of those 2 types of antibodies are associated. However, further studies examining Omicron-specific antibodies may reveal more subtle findings. A second limitation was that symptoms and disease severity were self-reported based on a questionnaire, which used both subjective and objective criteria. Another limitation is that adherence to the symptom survey was only 58% among HCWs who had test results positive for SARS-CoV-2 infection. However, our previous studies, in which adherence to symptom surveys was higher, found a similar distribution of symptoms,^6^ suggesting that the results were not biased in a specific direction. In addition, our infectivity analysis used N-gene Ct values as a correlate of infectivity, although this parameter is known to change throughout the duration of the infection and thus may not accurately represent infectivity. Furthermore, our cohort did not have any participants with severe disease or hospitalizations, and the threshold for protection from severe disease might be much lower.

## Conclusions

In this cohort study of vaccinated HCW, we found that a high humoral immune response, regardless of the number of booster doses or time since vaccination, was associated with reduced odds of Omicron infection and Omicron symptomatic disease. Thus, serological markers could be useful in assessing the risk of infection at both an individual and population level and in determining the necessity and timing of booster vaccination policies, even in the era of Omicron VOC.

## Notes

Supplement 1. **eMethods 1. **Variables in Protection From Infection Analysis **eMethods 2. **Variables in Protection From Symptomatic Disease Analysis **eMethods 3. **Serology Studies **eTable 1. **Variable Definitions **eTable 2. **Symptom Survey **eTable 3. **Study Population Characteristics of Participants Included in the Symptomatic Disease Analysis at their Exposure Event **eTable 4. **Study Population Characteristics of Participants Included in the Infectivity Analysis at Their Exposure Event **eTable 5. **Crude Odds Ratio of Infection By IgG Levels and Neutralizing Antibodies Levels **eTable 6. **Multivariable Logistic Regression Model for Factors Associated With Protection From Infection **eTable 7. **Baseline Characteristics of Survey Responders and Nonresponders **eTable 8. **Breakdown of Symptoms and Severity of Disease **eTable 9. **Crude Odds Ratio of Substantial Disease By IgG Levels and Neutralizing Antibodies Levels **eTable 10. **Binominal Negative Regression Model for Factors Associated With Protection From Symptomatic Disease **eTable 11. **Multivariable Logistic Regression Model for Factors Associated With Protection From Infectivity **eFigure 1.** Variant Circulation in Israel During the Study Period **eFigure 2.** Association of IgG and Neutralizing Antibodies Titers With Number of Days of Substantial Disease and Existence of Fever **eFigure 3.** Association of IgG and Neutralizing Antibody Titers With Ct Values

Supplement 2.
Data Sharing Statement
