# Supplementary material for: Factors Associated With Protection From SARS-CoV-2 Omicron Variant Infection and Disease Among Vaccinated Health Care Workers in Israel
Source: JAMA Netw Open. 2023 May 23;6(5):e2314757. doi: 10.1001/jamanetworkopen.2023.14757 (PMC10208153; doi:10.1001/jamanetworkopen.2023.14757)
Supplement: Supplement 1. — eMethods 1. Variables in Protection From Infection Analysis eMethods 2. Variables in Protection From Symptomatic Disease Analysis eMethods 3. Serology Studies eTable 1. Variable Definitions eTable 2. Symptom Survey eTable 3. Study Population Characteristics of Participants Included in the Symptomatic Disease Analysis at their Exposure Event eTable 4. Study Population Characteristics of Participants Included in the Infectivity Analysis at Their Exposure Event eTable 5. Crude Odds Ratio of Infection By IgG Levels and Neutralizing Antibodies Levels eTable 6. Multivariable Logistic Regression Model for Factors Associated With Protection From Infection eTable 7. Baseline Characteristics of Survey Responders and Nonresponders eTable 8. Breakdown of Symptoms and Severity of Disease eTable 9. Crude Odds Ratio of Substantial Disease By IgG Levels and Neutralizing Antibodies Levels eTable 10. Binominal Negative Regression Model for Factors Associated With Protection From Symptomatic Disease eTable 11. Multivariable Logistic Regression Model for Factors Associated With Protection From Infectivity eFigure 1. Variant Circulation in Israel During the Study Period eFigure 2. Association of IgG and Neutralizing Antibodies Titers With Number of Days of Substantial Disease and Existence of Fever eFigure 3. Association of IgG and Neutralizing Antibody Titers With Ct Values [file jamanetwopen-e2314757-s001.pdf]

Supplemental Online Content

Gilboa M, Gonen T, Barda N, et al. Factors associated with protection from SARS-CoV-2 Omicron variant infection and disease among vaccinated health care workers in Israel. *JAMA Netw Open.* 2023;6(5):e2314757. doi:10.1001/jamanetworkopen.2023.14757

eMethods 1. Variables in Protection From Infection Analysis

eMethods 2. Variables in Protection From Symptomatic Disease Analysis

eMethods 3. Serology Studies

eTable 1. Variable Definitions

eTable 2. Symptom Survey

eTable 3. Study Population Characteristics of Participants Included in the Symptomatic Disease Analysis at their Exposure Event

eTable 4. Study Population Characteristics of Participants Included in the Infectivity Analysis at Their Exposure Event

eTable 5. Crude Odds Ratio of Infection By IgG Levels and Neutralizing Antibodies Levels

eTable 6. Multivariable Logistic Regression Model for Factors Associated With Protection From Infection

eTable 7. Baseline Characteristics of Survey Responders and Nonresponders

eTable 8. Breakdown of Symptoms and Severity of Disease

eTable 9. Crude Odds Ratio of Substantial Disease By IgG Levels and Neutralizing Antibodies Levels

eTable 10. Binominal Negative Regression Model for Factors Associated With Protection From Symptomatic Disease

eTable 11. Multivariable Logistic Regression Model for Factors Associated With Protection From Infectivity

eFigure 1. Variant Circulation in Israel During the Study Period

eFigure 2. Association of IgG and Neutralizing Antibodies Titers With Number of Days of Substantial Disease and Existence of Fever

eFigure 3. Association of IgG and Neutralizing Antibody Titers With Ct Values

© 2023 Gilboa M et al. *JAMA Network Open.*

This supplemental material has been provided by the authors to give readers additional information about their work.

© 2023 Gilboa M et al. *JAMA Network Open.*

eMethods 1. Variables in Protection From Infection Analysis

Inclusion criteria: HCW who received 3 or 4 doses of the BNT162b2 vaccine; HCW who were tested at least once during the study period for SARS-CoV-2 via RT-PCR and whose sample was processed at SMC; HCW who provided at least one sera sample between December 2021 and May 2022 as part of the Sheba HCW serology cohort, and whose sera sample was collected not more than 30 days before a PCR test.

Exclusion criteria: HCW who had been diagnosed with a SARS-CoV-2 infection before January 2021 by RT-PCR or by serology testing (see supplementary for definition); HCW who had received only 1 or 2 doses of the Pfizer vaccine; those boosted with Moderna vaccine. HCW who had received a booster dose between serological test and PCR test.

Exclusion of serology tests due to previous infections:

Sera samples from HCW who were previously diagnosed with a SARS-CoV-2 infection in a non-study setting (whether RT-PCR or antigen testing) during the study's period were censored if they were obtained after such a diagnosis.

Additionally, HCWs who had an increase in IgG levels at >30 days after receiving either the 3rd/4th vaccine dose were excluded from the analysis based on the assumption that they had an unidentified SARS-CoV-2 infection. An increase in IgG levels was defined as follows:

1. If baseline serology values were <5000 BAU - an increase of >1000 BAU was determined as significant.
2. If baseline serology values were 5,000-15,000 BAU - an increase of >2,000 BAU was determined as significant.

© 2023 Gilboa M et al. *JAMA Network Open.*

eMethods 2. Variables in Protection From Symptomatic Disease Analysis

Inclusion criteria: SARS-COV-2 positive participants who answered at least one survey, and data on the severity of symptoms was available. Criteria for inclusion and exclusion of the serology tests were similar as above (regarding timing from testing and booster dose).

© 2023 Gilboa M et al. *JAMA Network Open.*

eMethods 3. Serology Studies

Serology assays

Samples from vaccinated participants were tested using the SARS-CoV-2 IgG II Quant (6S60, Abbott) test according to the manufacturer's instructions.

A SARS-CoV-2 pseudovirus (psSARS-2) neutralization assay was performed using a propagation-competent vesicular stomatitis virus spike shown to be highly correlative to authentic SARS-CoV-2 virus microneutralization assay. Following titration, 100 focus-forming units of psSARS-2 were incubated with twofold serial dilution of heat-inactivated (56 °C for 30 min) tested sera. After incubation for 60 min at 37 °C, virus/serum mixture was transferred to Vero E6 cells (CRL-1586, ATCC) that have been grown to confluency in 96-well plates and incubated for 90 min at 37 °C. After the addition of 1% methylcellulose (M0512, Sigma-Aldrich) in Dulbecco's modified Eagle's medium (Biological Industries) with 2% of fetal bovine serum (Biological Industries), plates were incubated for 24 h, and 50% plaque reduction titer was calculated by counting green fluorescent foci using a fluorospot reader (AID Autoimmun Diagnostika). Sera not capable of reducing viral replication by 50% at 1:8 dilution or below were considered nonneutralizing. For clear presentation, non-neutralizing samples were marked as a titter of 2.

© 2023 Gilboa M et al. *JAMA Network Open.*

eTable 1. Variable Definitions

Variable	Values	Definitions	Timing
**Outcomes**			
**Infection**	0/1		
**N Gene CT Value**	Continuous		
**Symptomatic disease**			
**Non-negligible symptoms**	0/1	at least one day of fever above 38, or at least one day of symptoms requiring to spend most of the day in bed	
**days with substantial symptoms**	count	days with symptoms requiring to spend most time in bed	
**days with a fever over 38 degrees Celsius**	count		
**the existence of fever over 38 degrees Celsius**	(binary),		
**symptomatic disease**	(binary)	reporting any symptoms	
**the existence of two or more days with substantial symptoms**	(binary),		
**the existence of three or more days with substantial symptoms (),**	binary		
**Variables**			
**IgG titer**	Continuous (S/CO)	SARS-CoV-2 Receptor Binding Domain (RBD) Immunoglobulin G (IgG) assay (Beckman-Coulter, CA, U.S.A.)	3-30 days before SARS-COV-2 PCR test
**NeutAb**	Continuous (50% titer)	SARS-CoV-2 Pseudo-virus (psSARS-2) Neutralization Assay	3-30 days before SARS-COV-2 PCR test
**Sex**	Female/male	As defined in SMC' files	Current
**Age**	Continuous (years)	As defined in SMC' files	At third vaccine dose

© 2023 Gilboa M et al. *JAMA Network Open.*

**Number of vaccines**	Categorical	As defined in SMC' files	Before PCR-testing event
**Time since last vaccine**	Continuous	As defined in SMC' files	Before PCR testing event
**BMI**	Categorical: <25, 25-29.99, ≥30	BMI was calculated by weight (kg)/(height (m))^2 according to the HCW answer to the questionnaire.	At the second vaccine dose
**Blood pressure disease**	0/1	According to the HCW answer to the questionnaire: defined as systolic blood pressure above 140 treated with medication	At the second vaccine dose
**Dyslipidemia**	0/1	According to the HCW answer to the questionnaire: defined as total cholesterol above 200 or LDL cholesterol above 160 treated with medication	At the second vaccine dose
**Autoimmune disease**	0/1	According to the HCW answer to the questionnaire: defined as a known autoimmune disease treated with medication	At the second vaccine dose
**Diabetes**	0/1	According to the HCW answer to the questionnaire: defined as HbA1C>6.5 or fasting blood sugar>126 treated with medication	At the second vaccine dose
**Heart disease**	0/1	According to the HCW answer to the questionnaire: defined as known heart disease treated with medication	At the second vaccine dose
**Lung disease**	0/1	According to the HCW answer to the questionnaire: defined as	At the second vaccine dose

© 2023 Gilboa M et al. *JAMA Network Open.*

		known lung disease treated with medication	
**Coagulation disorder**	0/1	According to the HCW answer to the questionnaire: defined as known hemorrhage or thrombosis disease treated with medication	At the second vaccine dose
**Immunosuppressed**	0/1	According to the HCW answer to the questionnaire: defined as organ transplantation, biologic therapy, chemotherapy, steroids, splenectomy, or HIV	At the second vaccine dose
**Allergy**	0/1	According to the HCW answer to the questionnaire: defined as a serious allergic reaction (anaphylaxis) that required immediate treatment	During the life
**Liver disease**	0/1	According to the HCW answer to the questionnaire: defined as cirrhosis, hepatitis, liver cancer, metabolic disorder	At the second vaccine dose
**Kidney disease**	0/1	According to the HCW answer to the questionnaire: defined as creatinine>1.2 or GFR<60) treated with medication	At the second vaccine dose

eTable 2. Symptom Survey

	Question	Response
1.	What was the highest fever measurement in the past 24 hours?	Under 37
		37-37.4
		37.5-37.9
		38-38.9
		39-39.9
		Over 40
2.	What was the lowest saturation measured in the past 24 hours?	Over 96

© 2023 Gilboa M et al. *JAMA Network Open.*

		Under 96
		Not measured
3.	**Please mark all symptoms that you experienced in the past 24 hours:**	Cough
		Dyspnea
		Myalgia
		Throat pain
		Headache
		Chest pain
		Fatigue
		Rhinorrhea
		Diarrhea
		Anosmia
		Other- please elaborate
		I did not experience any symptom
4.	**How would you describe your functional status**	I am hospitalized
		I cannot get out of bed
		I feel unwell and spend most day in bed
		I feel less well than usual
		I feel well as usual

© 2023 Gilboa M et al. *JAMA Network Open.*

eTable 3. Study Population Characteristics of Participants Included in the Symptomatic Disease Analysis at their Exposure Event

**Events included in the analysis**	**667**
**Age (median, IQR)**	46.28 (37.44,54.8)
**Male-sex**	151 (22.6%)
**Sector**	
**Administrative and maintenance**	162 (24.3%)
**Doctors**	110 (16.5%)
**Paramedical**	159 (23.8%)
**Nurses**	236 (35.4%)
**Number of background diseases**	(9.4% missing)
**0**	465 (77.6%)
**1**	98 (16.4%)
**2**	28 (4.7%)
**3**	7 (1.2%)
**4**	0 (0%)
**5**	1 (0.2%)
**Immunosuppression**	3 (0.5%)
**Number of previous vaccines**	
**Unvaccinated**	4 (0.6%)
**Partially vaccinated (1-2 doses)**	22 (3.2%)
**3 vaccines**	482 (72.3%)
**4 vaccines**	159 (23.8%)
**Time since the last vaccine (median [IQR])**	153 (98,168)
**IgG levels, BAU (median [IQR])**	871.8 (463,1569)
**IgG levels by category, BAU**	
**1-500**	180 (27%)
**501-900**	166(24.9%)
**901-1600**	157 (23.5%)
**>1600**	164 (24.6%)
**Neutralizing antibodies (median, IQR)**	1024 (256,2048)
**Neutralizing antibody titer by category**	
**1-256**	122 (25.7%)
**512-1024**	204 (42.9%)
**2048**	67 (14.1%)
**>2048**	82 (17.3%)

© 2023 Gilboa M et al. *JAMA Network Open.*

eTable 4. Study Population Characteristics of Participants Included in the Infectivity Analysis at Their Exposure Event

**Events included in the analysis**	**532**
**Age (median, IQR)**	48 (39, 56)
**Male sex (%)**	129 (24%)
**Number of previous vaccines**	
**3 vaccines**	410 (77%)
**4 vaccines**	122 (23%)
**Time since the last vaccine**	148 (103, 161)
**IgG levels, BAU (median [IQR])**	918 (489, 1,703)
**IgG levels by category, BAU**	
**0-500**	137 (26%)
**501-1000**	151 (28%)
**1001-2000**	139 (26%)
**>2000**	105 (20%)
**Neutralizing antibodies (median, IQR)**	1,024 (256, 2,048)
**Participants with Missing Neutralizing antibodies**	184
**Neutralizing antibody titer by category**	
**0-512**	141 (27%)
**1024-2048**	289 (54%)
**>2048**	102 (19%)
**N-Gene CT level (median, IQR)**	25.6 (21.3, 31.4)
**Missing CT-Value (Excluded)**	18

© 2023 Gilboa M et al. *JAMA Network Open.*

eTable 5. Crude Odds Ratio of Infection By IgG Levels and Neutralizing Antibodies Levels

		OR [95%CI]
IgG levels*(BAU)	501-1000	0.98 [0.77-1.25]
	1001-2000	0.86 [0.67-1.1]
	>2000	0.52 [0.39-0.67]
Neutralizing Antibodies levels** (titer)	1024	0.79[0.59-1.06]
	2048-4096	0.50[0.38-0.66]
	>4096	0.34[0.21-0.53]

* Compared to <500 BAU

** Compared to <1024

© 2023 Gilboa M et al. *JAMA Network Open.*

eTable 6. Multivariable Logistic Regression Model for Factors Associated With Protection From Infection

IgG Model	OR (95% CI)
Log10 IgG	0.71 (0.56-0.90)
Age	0.99 (0.98-1.0)
Male sex	1.41 (1.11-1.80)
4th dose vaccine	0.49 (0.22-1.10)
Time since last vaccine	1.00 (0.99-1.00)
Neutralizing antibodies Model	
Log2 Neutralizing antibodies	0.89 (0.83-0.95)
Age	0.98 (0.97-1.0)
Male sex	1.23 (0.88-1.72)
4th dose vaccine	0.11 (0.03-0.45)
Time since last vaccine	0.99 (0.98-1.00)

© 2023 Gilboa M et al. *JAMA Network Open.*

eTable 7. Baseline Characteristics of Survey Responders and Nonresponders

		Survey nonresponders N=2308	Survey responders N=3243
	Age- mean [95%CI]	41.9 [41.4-42.4]	42.5 [42.0-42.9]
	Male -n (%)	662 (29%)	803 (25%)
Number of vaccines	2 vaccines or less	476 (21%)	499 (15%)
	3 vaccine doses	1646 (71%)	2350 (72%)
	4 vaccines doses	186 (8%)	394 (12%)
	Previously recovered	165 (7%)	208 (6%)
Sector	Administrative and maintenance	721 (31%)	817 (25%)
	Doctors	457 (20%)	586 (18%)
	Paramedical	494 (21%)	724 (22%)
	Nurses	636 (28%)	1116 (34%)

© 2023 Gilboa M et al. *JAMA Network Open.*

eTable 8. Breakdown of Symptoms and Severity of Disease

Characteristics		N=3243	%
Number of days responding to survey	1	742	23%
	2	641	20%
	3	697	21%
	4	799	25%
	5 or more	364	11%
Maximal measured temperature	Under 38	2759	85%
	Over 38	484	15
Number of reported symptoms	0	331	10%
	1	264	8%
	2	350	11%
	3	450	14%
	4	509	16%
	5 or more	1339	41%
Days with symptoms	0	331	10%
	1	736	23%
	2	678	21%
	3	613	19%
	4	634	20%
	5 or more	251	7%
Degree of symptoms	No functional impairment	544	17%
	Slight functional impairment	1233	38.%
	Severe functional impairment	1350	42%
	Very severe functional impairment	116	4%
Days of substantial symptoms	0	1777	55%
	1	690	21%
	2	415	13%
	3	217	7%
	4	111	3%
	5	33	1%
Symptoms	Cough	2248	69%
	Rhinorrhea	22	69%
	Fatigue	2073	64%
	Headache	1950	60%
	Pharyngitis	1705	53%
	Myalgia	1645	51%
	Chest pain	381	12%
	Other	359	11%
	Anosmia	354	11%
	Dyspnea	345	11%
	Diarrhea	292	9%

© 2023 Gilboa M et al. *JAMA Network Open.*

eTable 9. Crude Odds Ratio of Substantial Disease By IgG Levels and Neutralizing Antibodies Levels

		OR [95%CI]
IgG levels*(BAU)	501-900	0.78 [0.51-1.20]
	901-1600	0.59 [0.38-0.90]
	>1600	0.43 [0.28-0.66]
Neutralizing Antibodies levels** (titer)	512-1024	0.71 [0.45-1.11]
	2048	0.48[0.26-0.89]
	>2048	0.38[0.21-0.67]

* Compared to <500 BAU

** Compared to <512

© 2023 Gilboa M et al. *JAMA Network Open.*

eTable 10. Binominal Negative Regression Model for Factors Associated With Protection From Symptomatic Disease

	Outcome	OR (95% CI)
The odds ratio for each 10-fold increase in IgG levels	Non-negligible symptoms	0.48 (0.29-0.78)
	Fever	0.70 (0.42-1.18)
	Increase in the number of Days with fever	0.82 (0.6-1.12)
	Increase in Maximal temperature measured	0.71 (0.46-1.14)
	Any symptomatic disease	0.72 (0.29-1.80)
	Days of substantial symptoms	0.82 (0.6-1.12)
	Two or more days with substantial symptoms	1.09 (0.64-1.86)
	Three or more days with substantial symptoms	1.12 (0.56-2.23)
The odds ratio for each 2-fold increase in Neutralizing antibodies levels	Non-negligible symptoms	0.86 (0.76-0.96)
	Fever	0.90 (0.78-1.04)
	Increase in the number of Days with fever	0.97 (0.88-1.06)
	Increase in Maximal temperature measured	0.89 (0.8-1.01)
	Any symptomatic disease	0.81 (0.64-1.03)
	Days of significant symptoms	0.94 (0.87-1.02)
	Two or more days with significant symptoms	0.92 (0.81-1.06)
	Three or more days with significant symptoms	0.95 (0.79-1.15)

All analyses were adjusted to age, gender, number of vaccines, time since the last vaccination, and number of background diseases. Male sex had a protective effect in all parameters with OR as low as 0.36 (95% CI 0.17-0.75). All other covariates for the adjustment that were measured were not significant.

© 2023 Gilboa M et al. *JAMA Network Open.*

eTable 11. Multivariable Logistic Regression Model for Factors Associated With Protection From Infectivity

IgG Model	OR (95% CI)
Log10 IgG	1.25 (-0.11-2.62)
Age	-0.06 (-0.10- -0.02)
Male sex	-0.76 (-2.0-0.51)
4th dose vaccine	-0.76 (-2.03-0.51)
Time from the last vaccine dose	-0.01 (-0.03-0.004)
Neutralizing antibodies Model	
Log2 Neutralizing antibodies	0.20 (-0.14- 0.55)
Age	-0.06 (-0.12-0.05)
Male sex	-0.51 (-2.05-1.04)
4th dose vaccine	-1.03 (-4.45-2.39)
Time from the last vaccine dose	-0.02 (-0.04-0.01)

© 2023 Gilboa M et al. *JAMA Network Open.*

eFigure 1. Variant Circulation in Israel During the Study Period

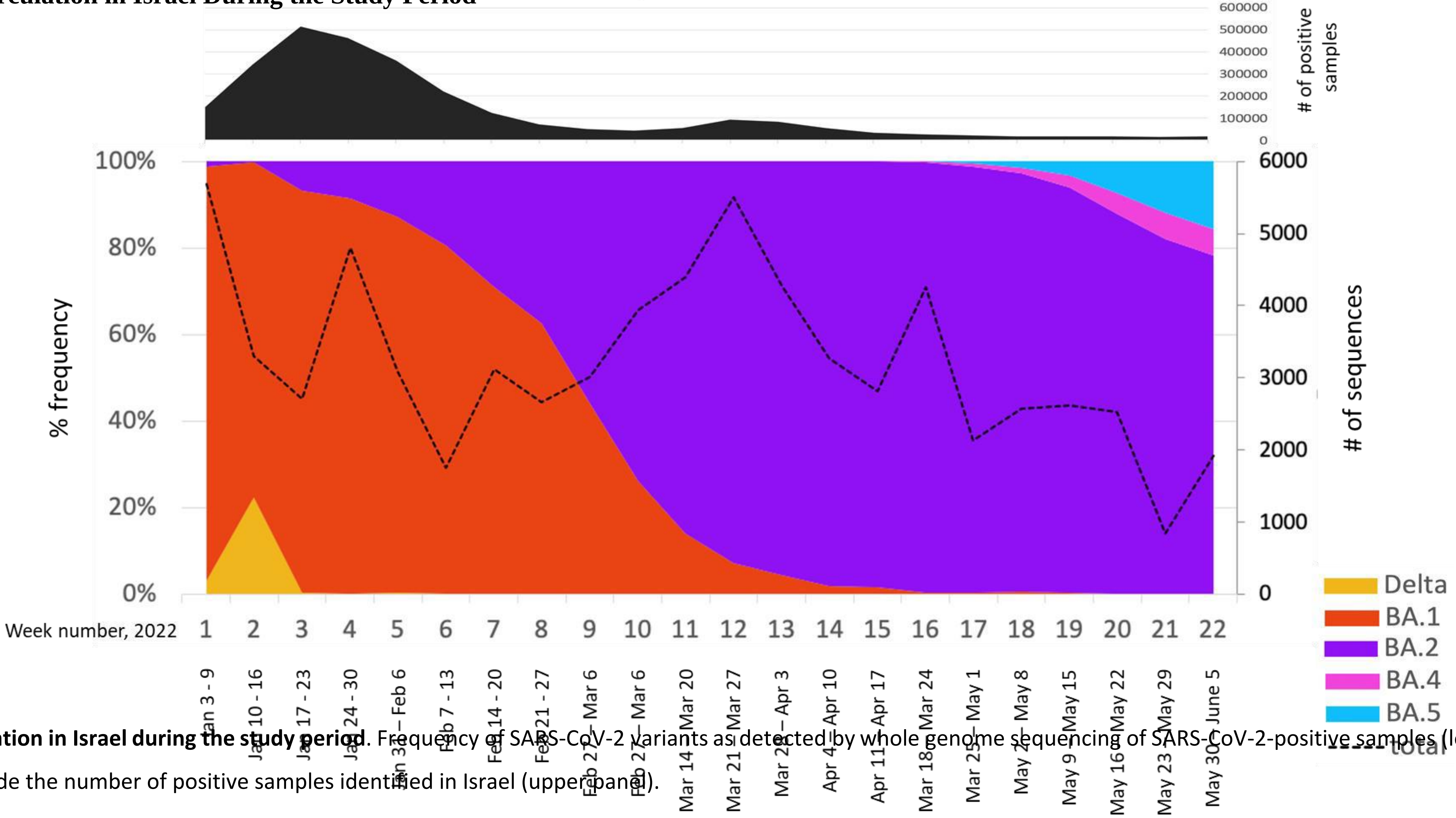

Variant circulation in Israel during the study period. Frequency of SARS-CoV-2 variants as detected by whole genome sequencing of SARS-CoV-2-positive samples (lower panel) alongside the number of positive samples identified in Israel (upper panel).

© 2023 Gilboa M et al. *JAMA Network Open.*

eFigure 2. Association of IgG and Neutralizing Antibodies Titers With Number of Days of Substantial Disease and Existence of Fever

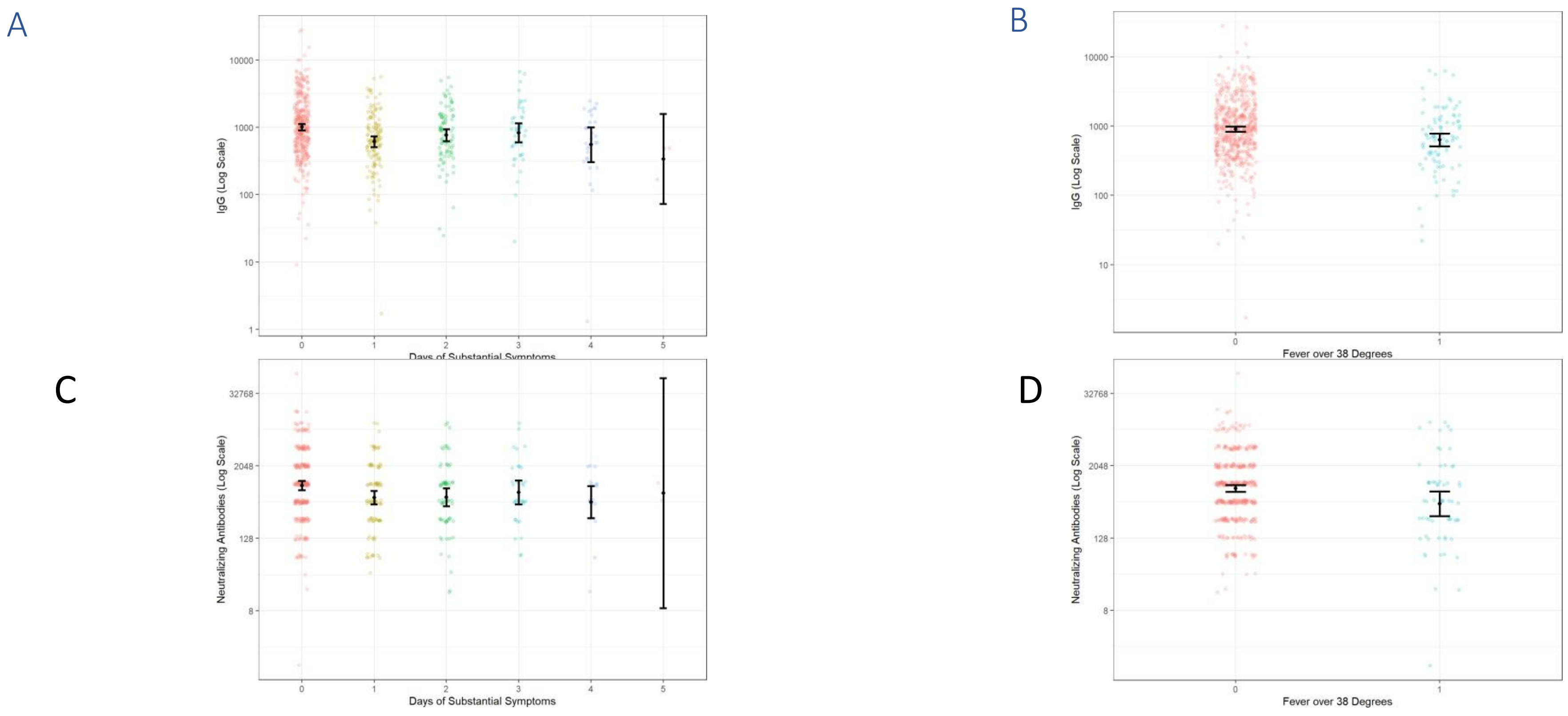

- Supplementary figure 1 Correlation of antibody titers and severity of disease- a. Number of days with substantial disease- days mostly bedridden and IgG antibody titers. B. Existence of fever (Above or below 30 degrees Celsius) and IgG antibody titers. c. Number of days with substantial disease- days mostly bedridden and Neutralizing antibody titers. d. Existence of fever (Above or below 30 degrees Celsius) and neutralizing antibody titers

© 2023 Gilboa M et al. *JAMA Network Open.*

eFigure 3. Association of IgG and Neutralizing Antibody Titers With Ct Values

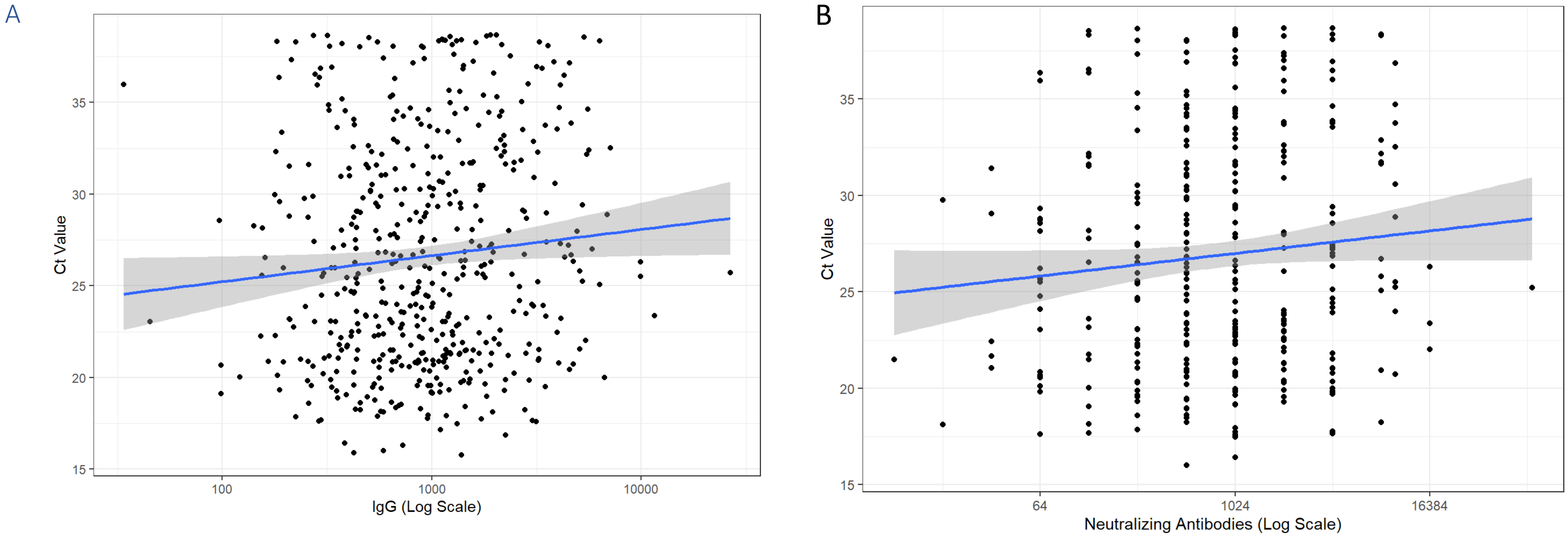

- Supplementary figure 2 Correlation of antibody titers and N gene CT (Cycle threshold) values as a representation of infectivity. Crude results of A. IgG antibody titers and B. Neutralizing antibodies titers with corresponding SARS-COV-2 RT PCR N gene CT Values. Blue line represents the linear regression

© 2023 Gilboa M et al. *JAMA Network Open.*
