# Supplementary material for: Factors Associated With Protection From SARS-CoV-2 Omicron Variant Infection and Disease Among Vaccinated Health Care Workers in Israel
Source: JAMA Netw Open. 2023 May 23;6(5):e2314757. doi: 10.1001/jamanetworkopen.2023.14757 (PMC10208153; doi:10.1001/jamanetworkopen.2023.14757)
Supplement: Supplement 2. — Data Sharing Statement [file jamanetwopen-e2314757-s002.pdf]

Data Sharing Statement

Gilboa. Factors Associated With Protection From SARS-CoV-2 Omicron Variant Infection and Disease Among Vaccinated Health Care Workers in Israel. *JAMA Netw Open*. Published May 23, 2023. doi:10.1001/jamanetworkopen.2023.14757

Data
Data available: Yes
Data types: Deidentified participant data
How to access data: De-identified clinical data for the patients in this study will be made available to other investigators after approval by the institutional review board. Requests should be directed to the corresponding author. gili.regev@sheba.health.gov.il
When available: With publication

Supporting Documents
Document types: None

Additional Information
Who can access the data: will be made available to other investigators after approval by the institutional review board.
Types of analyses: will be made available to other investigators after approval by the institutional review board.
Mechanisms of data availability: after approval of a proposal and signed data acsses agreement
